# Two Cases of Post‐Operative Coronary–Cameral Fistulas After Unusual Myectomy Procedures

**DOI:** 10.1002/ccr3.72862

**Published:** 2026-06-08

**Authors:** Neda Toofaninejad, Arezoo Hajiali, Mahtab Hatami, Saba Mohammadzadeh

**Affiliations:** ^1^ Department of Echocardiography, Tehran Heart Center, Cardiovascular Research Institute Tehran University of Medical Science Tehran Iran

**Keywords:** coronary artery fistula, double‐chambered RV, myectomy, postoperative complication, subaortic stenosis

## Abstract

Acquired coronary–cameral fistulas (CCFs) are rare complications characterized by abnormal communication between a coronary artery and a cardiac chamber. They may result from chest trauma, myocardial infarction, or iatrogenic injury during cardiac procedures, including percutaneous coronary intervention (PCI), pacemaker lead implantation, and cardiac surgery. We report two cases of post‐surgical CCF. The first case is a 50‐year‐old man with a double‐chambered RV who developed a small fistula to the right ventricular outflow tract (RVOT) following resection of the hypertrophied RV muscle bundle. The second case is a 41‐year‐old woman with a fistula from the basal interventricular septum into the left ventricular outflow tract (LVOT) after redo subaortic web resection and septal myectomy. Both fistulas were detected on postoperative echocardiography and were hemodynamically insignificant. Both patients were managed conservatively without intervention, consistent with the usual course of small post‐surgical CCFs. Post‐surgical CCFs are typically asymptomatic, often detected incidentally, and may persist or close spontaneously. Awareness of this complication and careful imaging are essential for appropriate follow‐up and management.

## Key Clinical Message

Small post‐surgical coronary–cameral fistulas can occur after various myectomy procedures. Although often asymptomatic and hemodynamically insignificant, they should be considered in the differential diagnosis of transventricular septal shunts, and their hemodynamic significance should be carefully assessed.

## Graphical abstract

Post‐surgical coronary–cameral fistulas (CCF) can occur after various myectomy procedures. Here we present one case of CCF after resection of the hypertrophied RV muscle bundle in double‐chambered RV (DCRV) and one case of CCF after subaortic web resection. *Key point*: Watch for diastolic trans–ventricular septal flows following surgical myectomies.

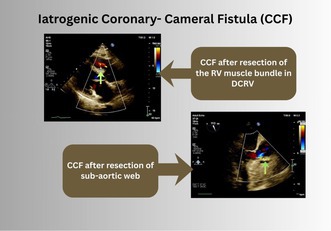

**Abbreviations**
AR: aortic regurgitation
ASD: atrial septal defect
CCF: coronary–cameral fistula
CT: computed tomography
DCRV: double‐chambered right ventricle
LVOT: left ventricular outflow tract
NYHA: New York Heart Association
PCI: percutaneous coronary intervention
PTE: percutaneous therapeutic embolization
PTPC: percutaneous pulmonary valve commissurotomy
RCC: right coronary cusp
RV: right ventricle
RVOT: right ventricular outflow tract
VSD: ventricular septal defect

## 1 Introduction

Acquired coronary–cameral fistula (CCF) is a very rare clinical entity characterized by an abnormal communication between a coronary artery and a cardiac chamber. Unlike congenital fistulas, which arise from developmental anomalies, the acquired form typically develops secondary to external injury or iatrogenic causes. Reported etiologies include blunt or penetrating chest trauma, acute myocardial infarction, percutaneous coronary interventions (PCI), pacemaker lead implantation, and various forms of cardiac surgery, all of which may disrupt the integrity of the coronary arterial wall and adjacent cardiac structures [1]. Although often asymptomatic, acquired coronary–cameral fistulas may lead to significant hemodynamic consequences depending on their size, flow characteristics, and the cardiac chamber involved [2].

## 2 Case 1

### 2.1 History and Physical Examination

A 50‐year‐old man with a remote history of percutaneous pulmonary valve commissurotomy (PTPC) presented with progressive dyspnea over the preceding 2 years. On physical examination, he was normotensive with an oxygen saturation of 92% on room air. Cardiovascular examination revealed a holosystolic 4/6 murmur at the lower left sternal border. Other systemic examinations were unremarkable.

### 2.2 Investigations, Differential Diagnosis

Transthoracic echocardiography demonstrated a prominent right ventricular outflow tract (RVOT) muscle bundle dividing the right ventricle (RV) into a high‐pressure proximal chamber and a low‐pressure distal chamber, consistent with double‐chambered right ventricle (DCRV). Doppler interrogation across the obstructive muscle bundle revealed severe intraventricular obstruction with a maximum pressure gradient of 68 mmHg.

**FIGURE 1:**
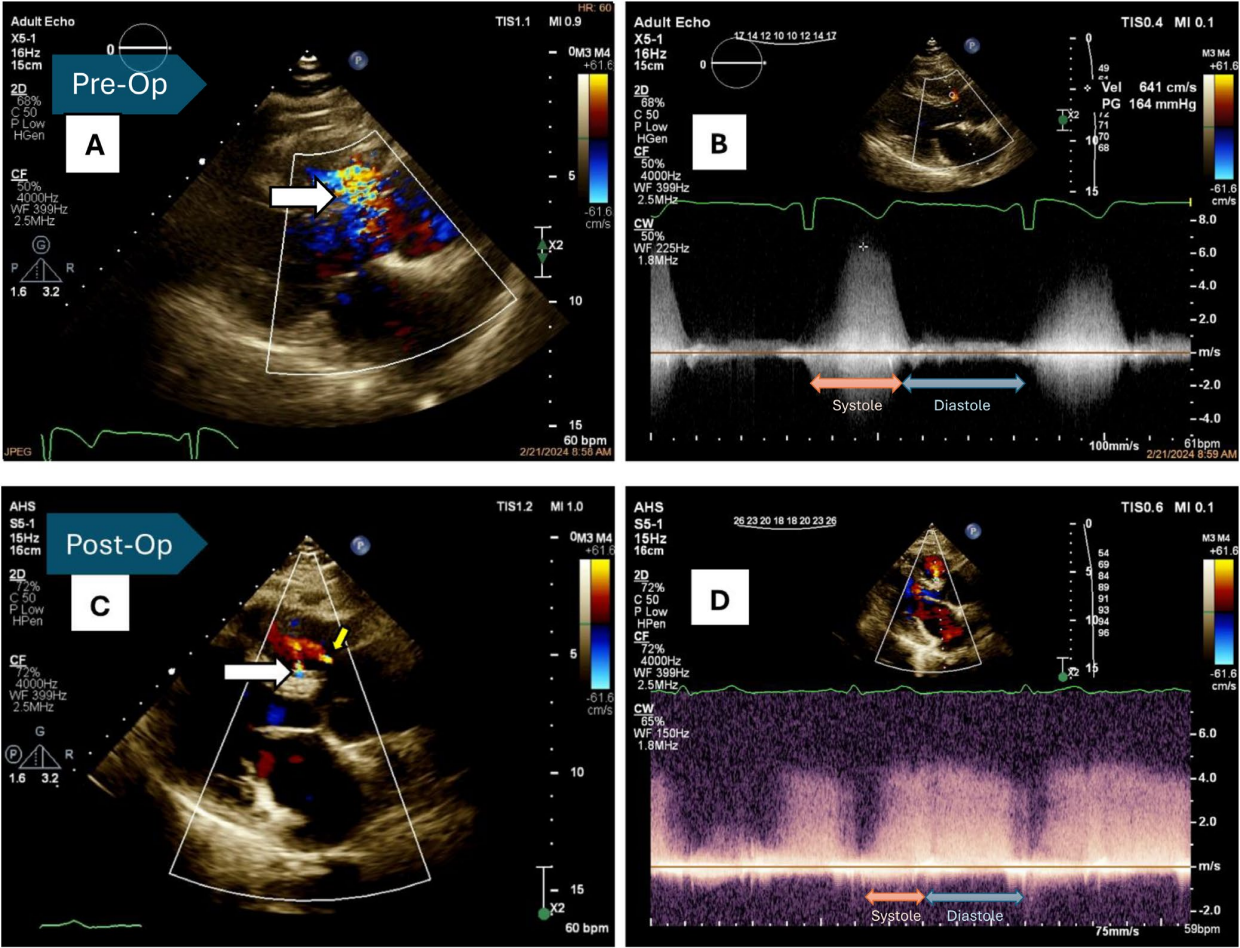
Transthoracic echocardiography; pre‐operative (A, B) and post‐operative (C, D). (A) *Pre‐operative* parasternal long‐axis view with color Doppler showing perimembranous VSD turbulent jet flow from interventricular septum, visible just below aortic valve. (B) Continuous‐wave Doppler tracing across the jet shows a predominantly systolic, high‐velocity flow pattern, consistent with VSD flow. (C) *Post‐operative* parasternal long‐axis view with color Doppler demonstrating the acquired coronary–cameral fistula, visualized as jet flow originating from the interventricular septum (lower than preoperative VSD jet flow visible in A) and directed toward the RVOT (white arrow). Pulmonary regurgitation diastolic flow is also observed in this view (yellow arrow). (D) Continuous‐wave Doppler tracing across the jet shows a continuous, predominantly diastolic high‐velocity flow pattern consistent with a coronary–cameral fistula.

Additional findings included a small (6 mm) perimembranous ventricular septal defect (VSD) with left‐to‐right shunt (Figure 1 and Video 1) and a secundum‐type atrial septal defect (ASD).

**VIDEO 1:** pre‐operative transthoracic echocardiography in parasternal long‐axis view with color Doppler showing perimembranous VSD jet flow from interventricular septum, visible just below aortic valve with mostly systolic flow, as well as turbulent flow caused by DCRV flow. Video content can be viewed at https://onlinelibrary.wiley.com/doi/10.1002/ccr3.72862.

Given the combination of significant RVOT obstruction and intracardiac shunts, the patient underwent surgical repair, including perimembranous VSD closure, resection of the hypertrophied RV infundibular muscle bundle with RVOT reconstruction, and closure of the secundum ASD.

Postoperative echocardiography showed complete closure of both the ASD and VSD, with no residual RVOT narrowing and no turbulent flow across the reconstructed outflow tract. However, color Doppler imaging demonstrated a new, small, high‐velocity continuous, predominantly diastolic flow jet (5 m/s) originating from the interventricular septum and directed toward the RVOT, consistent with iatrogenic coronary‐cameral fistula (Figure 1 and Video 2). LV had normal size with mild global systolic dysfunction (LVEF = 50%) like before surgery, and no new regional wall motion abnormalities (RWMA) were visualized. There was also mild pulmonary regurgitation and mild mitral regurgitation.

**VIDEO 2:** Post‐operative transthoracic echocardiography in parasternal long‐axis view with color Doppler demonstrating the acquired coronary–cameral fistula, visualized as jet flow originating from the interventricular septum and directed toward the RVOT. Pulmonary regurgitation diastolic flow is also observed in this view. Video content can be viewed at https://onlinelibrary.wiley.com/doi/10.1002/ccr3.72862.

Iatrogenic VSD is an important differential diagnosis for new post‐myectomy transseptal flows; however, the predominantly diastolic pattern of flow in our case was against this.

Table 1 presents a comparison of the pathologic jets shown in Figure 1.

**TABLE 1:** Comparison of the pathologic jets shown in Figure 1.

Pathologic lesion	Site of jet	Mechanism	Doppler phase	Course
CCF (post DCRV surgery)	IVS toward RVOT	Connection between coronary artery branches and RVOT	Continuous–predominantly diastolic	Usually hemodynamically insignificant
VSD	IVS toward RV	Defect in IVS	Systolic	Dependent on size
PR	PV toward RV	Abnormality in pulmonary valve	Diastolic	Dependent on severity

### 2.3 Treatment, Outcome and Follow Up

Our patient was asymptomatic, with no chest pain or dyspnea and had no hemodynamically significant shunt, as there was no chamber enlargement. There were no ischemic changes on ECG, and he was therefore managed conservatively. Considering the low risk for thrombosis, antithrombotic therapy was not initiated. No symptoms were reported by the patient during 12 months of follow‐up, and echocardiographic findings remained stable.

## 3 Case 2

### 3.1 History and Physical Examination

The second case is a 41‐year‐old woman with a history of subaortic web resection and septal myectomy performed 5 years earlier. Over the past year, she experienced worsening dyspnea (NYHA functional class III–IV) and orthopnea. Physical examination revealed normal blood pressure and a 4/6 systolic ejection murmur best heard at the left lower sternal border.

### 3.2 Investigations, Differential Diagnosis

**FIGURE 2:**
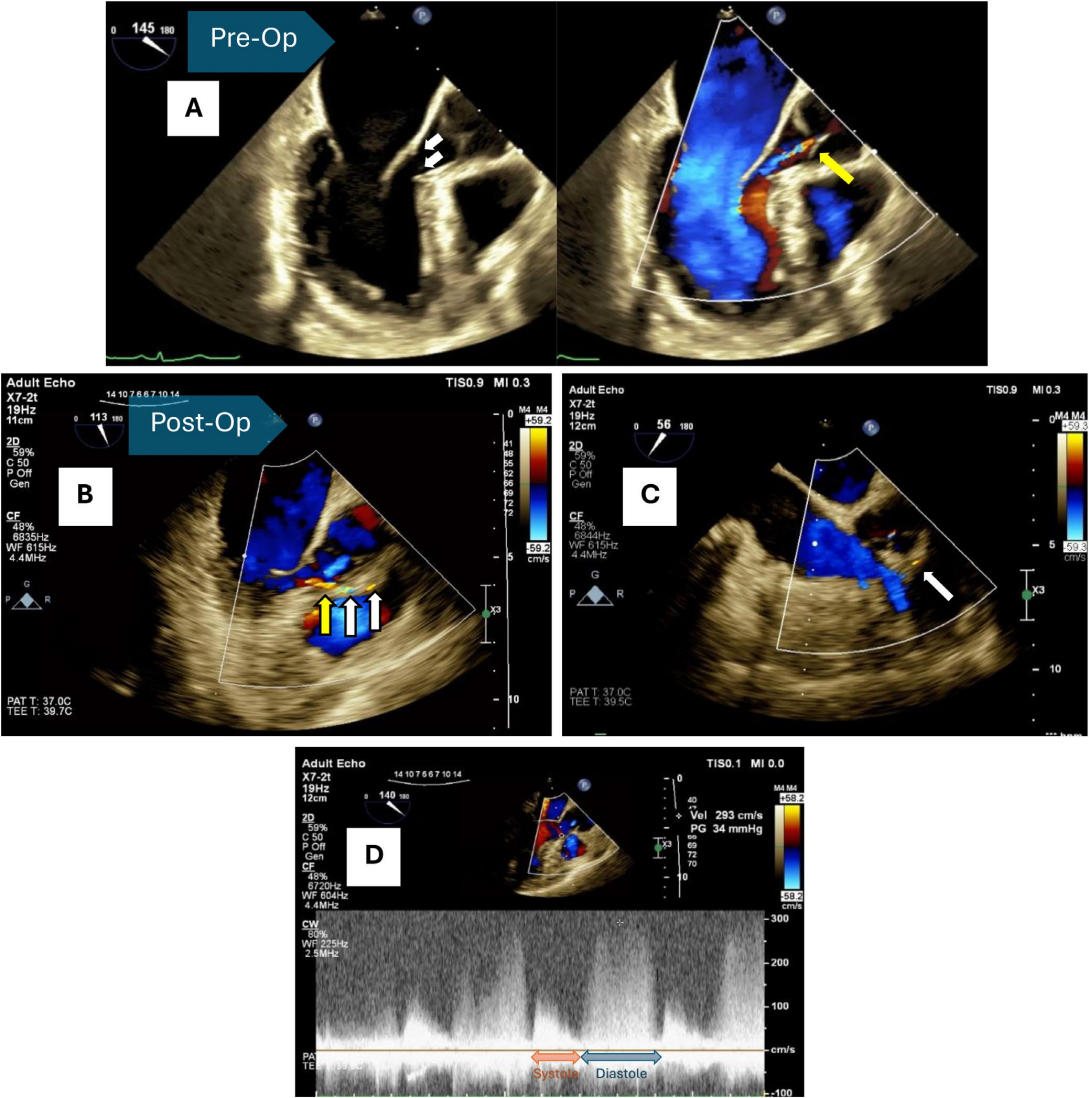
Transesophageal echocardiography; pre‐operative (A) and post‐operative (B–D). (A) *Preoperative* aortic long‐axis views without (left) and with (right) color Doppler showing a subaortic web (white arrows). The only diastolic flow is aortic regurgitation (yellow arrow), with no abnormal fistula flow. (B) *Postoperative* aortic long‐axis view with color Doppler demonstrating an abnormal diastolic flow from the basal interventricular septum into the left ventricular outflow tract (LVOT) (white arrow), with oblique orientation of the exit jet directed away from the interventricular septum (yellow arrow), consistent with coronary–cameral fistula. (C) *Postoperative* aortic short‐axis view with color Doppler showing a cross‐sectional image of the same fistulous tract arising from the basal interventricular septum in diastole (white arrow). (D) Continuous‐wave Doppler aligned with the jet, revealing diastolic flow (2.93 m/s).

Echocardiography demonstrated recurrent narrowing of the left ventricular outflow tract (LVOT) with turbulent flow and a significantly elevated LVOT gradient (peak pressure gradient: 34 mmHg, mean pressure gradient: 22 mmHg), findings consistent with recurrence of a subaortic web (Figure 2; Videos 3 and 4). Severe tricuspid regurgitation was also detected. The patient subsequently underwent redo subaortic web resection, repeat myectomy, and tricuspid valve replacement with a bioprosthetic valve. Postoperative transesophageal echocardiography showed an abnormal diastolic flow from the basal interventricular septum into the left ventricular outflow tract consistent with a coronary–cameral fistula caused by trauma to the septal perforator of the left anterior descending coronary artery during myectomy (Figure 2; Videos 5 and 6). The left ventricle was normal in size with preserved systolic function and no regional wall motion abnormalities. Mild aortic insufficiency was noted and the bioprosthetic tricuspid valve demonstrated acceptable gradients without regurgitation.

**VIDEO 3:** Preoperative TEE in aortic long‐axis view in gray scale showing a subaortic web. Video content can be viewed at https://onlinelibrary.wiley.com/doi/10.1002/ccr3.72862.

**VIDEO 4:** Preoperative TEE in aortic long‐axis view with color Doppler showing systolic turbulent flow passing subaortic web. The only diastolic flow is aortic regurgitation, with no abnormal fistula flow. Video content can be viewed at https://onlinelibrary.wiley.com/doi/10.1002/ccr3.72862.

**VIDEO 5:** Post‐operative TEE in aortic long‐axis view with color Doppler demonstrating an abnormal diastolic flow from the basal interventricular septum into the left ventricular outflow tract (LVOT), with oblique orientation of the exit jet directed away from the interventricular septum consistent with coronary–cameral fistula. Video content can be viewed at https://onlinelibrary.wiley.com/doi/10.1002/ccr3.72862.

**VIDEO 6:** Post‐operative TEE in aortic short‐axis view with color Doppler showing a cross‐sectional image of the coronary‐cameral fistula arising from the basal interventricular septum in diastole. Video content can be viewed at https://onlinelibrary.wiley.com/doi/10.1002/ccr3.72862.

An important differential diagnosis for post septal myectomy abnormal flow is ventricular septal defect (VSD) caused by defect formation in the interventricular septum. The VSD flow jet is systolic with a left‐to‐right shunt, which distinguishes it from CCF.

Aortic regurgitation (AR) can present with a diastolic flow pattern similar to that of a CCF, making it an important differential diagnosis. However, in this case, the presence of a visible tract adjacent to the aortic sinus, along with the oblique orientation of the exit jet directed away from the interventricular septum, helps distinguish it from the flow of AR [3].

### 3.3 Treatment, Outcome and Follow Up

The patient did not report any dyspnea or ischemic symptoms; there wasn't any chamber enlargement or new wall motion abnormalities. ECG didn't show any ischemic changes as well; therefore, a conservative management approach was planned. Given the low risk of thrombosis or distal embolization, no antithrombotic therapy was administered. At 12‐month follow‐up, the patient reported no new symptoms, and echocardiographic findings remained unchanged.

## 4 Discussion

Coronary‐cameral fistulas are rare. They are most often identified incidentally during coronary angiography or echocardiography. Approximately 35% of reported cases are acquired [1]. Acquired CCFs can develop as a complication of cardiac surgery due to iatrogenic injury to the coronary artery or myocardial wall, resulting in an abnormal communication with a cardiac chamber.

Most previously reported post‐surgical CCFs occur after post‐septal myectomy in patients with hypertrophic cardiomyopathy (HCM), with lower prevalence following coronary artery bypass grafting, aortic or mitral valve surgery, or surgical correction of congenital heart defects [4]. Post septal myectomy coronary cameral fistula occurs in about 19%–23% of myectomies as detected by echocardiographic follow‐up 1 month after surgery [4, 5]. In this setting, fistulas are thought to result from injury to septal perforator branches embedded within a hypertrophied interventricular septum during deep myectomy. Younger age, a thicker septum, and a deeper myectomy are risk factors for CCF formation after myectomy [2].

In contrast, we report two cases of post‐surgical CCFs occurring after less commonly described procedures: double‐chambered right ventricle (DCRV) repair and redo subaortic web resection with septal myectomy. Both cases were incidentally identified on postoperative echocardiography and were hemodynamically insignificant, managed conservatively with stable 12‐month follow‐up.

The underlying mechanisms in these two settings appear to differ from classic HCM‐related fistulas. In the DCRV case, the most possible mechanism is injury to the circle of Vieussens, an arterial network connecting the conal branch of the right coronary artery and a branch of the left anterior descending artery that supplies the right ventricular infundibulum. Surgical resection of hypertrophied infundibular muscle bundles or ventriculotomy may disrupt this vascular plexus, allowing direct communication between coronary microcirculation and the right ventricular outflow tract (RVOT). As a result, blood from either the right or left coronary circulation can enter directly into the right ventricular cavity, forming a small coronary–cameral communication [6]. This mechanism reflects the unique vascular anatomy of the RV infundibulum and differs fundamentally from septal perforator injury seen in HCM.

To our knowledge, only one previous case of CCF following DCRV surgery has been reported. Similar to our observation, the fistula was detected on postoperative echocardiography as a low‐velocity, predominantly diastolic flow without hemodynamic significance. However, the absence of long‐term follow‐up in that report limits understanding of its natural history [6].

In the second case, involving subaortic web resection and septal myectomy, the fistula likely resulted from localized injury to small intramyocardial vessels within the basal interventricular septum during resection. Unlike HCM, where marked septal hypertrophy necessitates deep myocardial excision, subaortic web disease is characterized by a less hypertrophied and more fibrotic substrate. This may lead to a different depth of surgical dissection and predispose to superficial coronary–cameral communication into the left ventricular outflow tract (LVOT).

When compared with HCM‐related post‐myectomy fistulas, our cases highlight that CCFs are not a uniform entity but vary according to surgical indication, myocardial substrate, and coronary anatomy. Table 2 summarizes these differences, demonstrating variations in fistula location and underlying mechanism while sharing a similar benign clinical course [4].

**TABLE 2:** Comparison of common post‐septal myectomy coronary–cameral fistulas in HCM with our two reported cases.

Case report	Surgery type	Fistula site	Doppler phase	Clinical course/outcome
Most prior reports	HCM, septal myectomy	Septal perforator branches to LV cavity	Predominant diastolic	Majority asymptomatic and disappeared spontaneously (78%)
Case 1	DCRV muscle bundle resection	Coronary branches to RV	Continuous‐ predominantly diastolic	Small, hemodynamically insignificant, conservative
Case 2	Redo subaortic web resection	Coronary branches to LV	Diastolic	Small, hemodynamically insignificant, conservative

Despite these mechanistic differences, the clinical behavior of post‐surgical CCFs appears consistent. Most are small, incidentally detected, and hemodynamically insignificant.

Small post‐septal myectomy CCFs are usually asymptomatic and may close spontaneously; however, some studies report persistence for several years [1].

The management of CCFs is often conservative, as in our two cases. Percutaneous therapeutic embolization (PTE) is rarely required and is reserved for symptomatic patients [4, 7, 8]. The development of symptoms, a significant left‐to‐right shunt, or myocardial ischemia due to a steal phenomenon should prompt consideration for intervention [8, 9, 10, 11]. For patients managed conservatively, periodic surveillance with electrocardiography and transthoracic echocardiography is recommended to monitor shunt magnitude, chamber size, ventricular function, pulmonary pressures, and signs of ischemia. Because major guidelines do not define specific follow‐up intervals, surveillance should be individualized according to fistula size, chamber enlargement, symptoms, ventricular function, and evidence of ischemia. Based on expert opinion, small asymptomatic fistulas may be followed with echocardiography every 2–5 years, whereas closer follow‐up (e.g., every 6–12 months) may be considered in patients with borderline chamber enlargement or other clinical concerns. More frequent reassessment may be warranted in the setting of progressive dilation or new symptoms [10].

## 5 Conclusion

Our report adds to the limited literature on post‐surgical CCFs, which are predominantly reported following septal myectomy in hypertrophic cardiomyopathy (HCM). Notably, our cases occurred after resection of a double‐chambered right ventricle (DCRV) in one patient and subaortic web resection with septal myectomy in the other.

Awareness of this rare complication is important for postoperative follow‐up, and appropriate imaging is essential to guide management. Clinicians should consider conservative monitoring while remaining cautious of potential symptoms that may necessitate intervention.

## Author Contributions

**Neda Toofaninejad:** conceptualization, supervision, validation. **Saba Mohammadzadeh:** data curation, resources, writing – original draft, writing – review and editing. **Arezoo Hajiali:** resources. **Mahtab Hatami:** resources.

## Funding

The authors have nothing to report.

## Consent

Written informed consent was obtained from the patients for the publication of this case report, in accordance with the consent statement provided in the manuscript.

## Conflicts of Interest

The authors declare no conflicts of interest.

## Data Availability

The data that support the findings of this study are available on request from the corresponding author. The data are not publicly available due to privacy or ethical restrictions.
